# Layer-Skipping Connections Improve the Effectiveness of Equilibrium Propagation on Layered Networks

**DOI:** 10.3389/fncom.2021.627357

**Published:** 2021-05-17

**Authors:** Jimmy Gammell, Sonia Buckley, Sae Woo Nam, Adam N. McCaughan

**Affiliations:** ^1^National Institute of Standards and Technology, Boulder, CO, United States; ^2^Department of Electrical, Computer and Energy Engineering, University of Colorado Boulder, Boulder, CO, United States

**Keywords:** equilibrium propagation, deep learning, small-world, layer-skipping connections, neuromorphic computing, biologically-motivated

## Abstract

Equilibrium propagation is a learning framework that marks a step forward in the search for a biologically-plausible implementation of deep learning, and could be implemented efficiently in neuromorphic hardware. Previous applications of this framework to layered networks encountered a vanishing gradient problem that has not yet been solved in a simple, biologically-plausible way. In this paper, we demonstrate that the vanishing gradient problem can be mitigated by replacing some of a layered network's connections with random layer-skipping connections in a manner inspired by small-world networks. This approach would be convenient to implement in neuromorphic hardware, and is biologically-plausible.

## 1. Introduction

As research into neural networks grows, there has been increased interest in designing biologically-inspired training algorithms, as they may offer insight into biological learning processes and also offer clues toward developing energy-efficient neuromorphic systems (Lillicrap et al., 2014; Bengio et al., 2015; Bartunov et al., 2018; Wozniak et al., 2018; Crafton et al., 2019; Ernoult et al., 2020). The equilibrium propagation learning framework introduced in Scellier and Bengio (2016) is one such algorithm. It is a method for training a class of energy-based networks, the prototype for which is the continuous Hopfield network (Hopfield, 1984). In particular, it addresses one of the major issues that prevent other training algorithms (such as backpropagation) from being biologically-plausible, which is the requirement for separate computation pathways for different phases of training. This also makes the algorithm appealing for practical implementation into neuromorphic hardware, because only a single computation circuit is required within each (non-output) neuron, rather than multiple distinct circuits. However, current implementations of the algorithm still have a defect that diminishes its biological plausibility: they require hand-tuned per-layer hyperparameters to account for a vanishing gradient through the network. In addition to not being biologically plausible, these multiplicative hyperparameters would be difficult to implement in a neuromorphic hardware system with limited bit depth. In this work, we demonstrate that the vanishing gradient problem can instead be addressed through topological means: by randomly replacing some of a layered network's connections with layer-skipping connections, we can generate a network that trains each layer more evenly and does not need per-layer hyperparameters. This solution is biologically-plausible and would be easier to implement in a neuromorphic system; additionally, it entails hand-tuning only two new hyperparameters (the number of layer-skipping connections and their initial weights), whereas the original solution adds a new hyperparameter for each pair of layers in a network.

Implementation of equilibrium propagation in Scellier and Bengio (2016) was hindered by a vanishing gradient problem whereby networks with as few as three hidden layers trained slowly on MNIST (LeCun and Cortes, 1998)—a serious issue given that network depth is critical to performance on difficult datasets (Simonyan and Zisserman, 2014; Srivastava et al., 2015b) and that convergence to a low error rate on MNIST is a low bar to meet. The problem was overcome in Scellier and Bengio (2016) by independently tuning a unique learning rate for each layer in the network. These learning rates were multiplicative factors that proportionally scaled the signals communicated between layers.

In our work, we have modified the strictly-layered topology of the original implementation by adding and removing connections to create a small-world-like network (Watts and Strogatz, 1998). Through this modification we have eliminated the per-layer hyperparameters without substantially degrading the algorithm's performance—after 250 epochs the modified network produces 0.0117% training error (out of 50,000 examples) and 2.55% test error (out of 10,000 examples) on MNIST using a network with three hidden layers and no regularization term in its cost function. These error rates are comparable to those of other biologically-motivated networks (Bartunov et al., 2018) and are approximately the same as those of the layered network with unique, manually-tuned learning rates in Scellier and Bengio (2016). Our method could be implemented with relative ease in any system with configurable connectivity, such as those already described in several neuromorphic hardware platforms (Schemmel et al., 2010; Davies et al., 2018; Shainline et al., 2019). Layer-skipping connections have been observed in biological brains (Bullmore and Sporns, 2009), so the approach is biologically-plausible. Similar techniques have seen success in convolutional (He et al., 2015; Srivastava et al., 2015a) and multilayer feedforward (Xiaohu et al., 2011; Krishnan et al., 2019) networks. Our findings outlined in this paper suggest that layer-skipping connections are effective-enough to be appealing in contexts where simplicity and biological plausibility are important. While small-world networks are not a novel concept, to our knowledge our work is the first to train small-world-like networks using the Equilibrium Propagation learning framework.

## 2. Background

### 2.1. Equilibrium Propagation

Similarly to backpropagation, the equilibrium propagation algorithm (Scellier and Bengio, 2016) trains networks by approximating gradient descent on a cost function. Equilibrium propagation is applicable to any network with dynamics characterized by evolution to a fixed point of an associated energy function; our implementation is a recreation of that in Scellier and Bengio (2016), which applies it to a continuous Hopfield network (Hopfield, 1984). The mathematical formulation of the framework can be found in Scellier and Bengio (2016). We discuss its appeal over backpropagation in section 4.2.

#### 2.1.1. Implementation in a Continuous Hopfield Network

Here we summarize the equations through which a continuous Hopfield network is trained using equilibrium propagation; this summary is based on the more-thorough and more-general treatment in Scellier and Bengio (2016).

Consider a network with *n* neurons organized into an input layer with *p* neurons, hidden layers with *q* neurons and an output layer with *r* neurons. Let the activations of these neurons be denoted, respectively by vectors **x** ∈ **ℝ**^*p*^, **h** ∈ **ℝ**^*q*^ and **y** ∈ **ℝ**^*r*^, and let **s** = (**h**^*T*^, **y**^*T*^)*T* ∈ **ℝ**^*q*+*r*^ and **u** = (**x**^*T*^, **s**^*T*^)*T* ∈ **ℝ**^*n*^ be vectors of, respectively, the activations of non-fixed (non-input) neurons and of all neurons in the network. Let **W** ∈ **ℝ**^*n*×*n*^ and **b** ∈ **ℝ**^*n*^ denote the network's weights and biases where *w*_*ij*_ is the connection weight between neurons *i* and *j* and *b*_*i*_ is the bias for neuron *i* (∀*i* *w*_*ii*_ = 0 to prevent self-connections), and let ρ denote its activation function; here and in Scellier and Bengio (2016),

(1) $$\rho (x)=\{\begin{array}{ll} 0 & x<0\\ x & 0\leq x\leq 1\\ 1 & x>1 \end{array}$$

is a hard sigmoid function where ρ′(0) = ρ′(1) is defined to be 1 to avoid neuron saturation. Let $\rho \left({\left(x_{1},\ldots ,x_{n}\right)}^{T}\right)={\left(\rho \left(x_{1}\right),\ldots ,\rho \left(x_{n}\right)\right)}^{T}$.

The behavior of the network is to perform gradient descent on a total energy function *F* that is modified by a training example (**x**_*d*_, **y**_*d*_). Consider energy function *E*:**ℝ**^*n*^ → **ℝ**,

(2) $$E(u;W,b)=\frac{1}{2}u^{T}u-\frac{1}{2}\rho {\left(u\right)}^{T}W\rho (u)-b^{T}u$$

and arbitrary cost function $C:{\mathbb{R}}^{r}\to {\mathbb{R}}_{+}$; here and in Scellier and Bengio (2016) it is a quadratic cost function given by

(3) $$C(y)=\frac{1}{2}||y-y_{d}|{|}_{2}^{2},$$

though the framework still works for cost functions incorporating a regularization term dependent on **W** and **b**. The total energy function *F*:**ℝ**^*n*^ → **ℝ** is given by

(4) F(u;β,W,b)=E(u;W,b)+βC(y)

where the clamping factor β is a small constant. **s** evolves over time *t* as

(5) $$\frac{ds}{dt}\propto -\frac{\partial F}{\partial s}.$$

Equilibrium has been reached when $\frac{\partial F}{\partial \text{s}}\approx 0$. This can be viewed as solving the optimization problem

(6) $$\underset{s\in {\mathbb{R}}^{q+r}}{\text{minimize}}\;F\left({\left(x_{d}^{T},s^{T}\right)}^{T};\beta ,W,b\right)$$

by using gradient descent to find a local minimum of *F*. Parameters θ can then be updated using the rule

(7) $$\Delta \theta \propto -\frac{1}{\beta }(\frac{\partial F}{\partial \theta }({\left(x_{d}^{T},s^{T}\right)}^{T};\beta ,W,b)-\frac{\partial F}{\partial \theta }({\left(x_{d}^{T},s^{T}\right)}^{T};0,W,b)).$$

The procedure for training on a single input-output pair (**x**_*d*_, **y**_*d*_) is as follows:

1. Clamp **x** to **x**_*d*_ and perform the free-phase evolution: evolve to equilibrium on the energy function *F*(**u**; 0, **W**, **b**) in a manner dictated by Equation (5). Record the equilibrium state **u**^0^.
2. Perform the weakly-clamped evolution: evolve to equilibrium on the energy function *F*(**u**; β, **W**, **b**) using **u**^0^ as a starting point. Record the equilibrium state **u**^β^.
3. Compute the correction to each weight in the network:
  (8) $$\Delta W_{ij}=\frac{1}{\beta }(\rho (u_{i}^{\beta })\rho (u_{j}^{\beta })-\rho (u_{i}^{0})\rho (u_{j}^{0})).$$
  Adjust the weights using *W*_*ij*_ ← *W*_*ij*_ + αΔ*W*_*ij*_ where the learning rate α is a positive constant.
4. Compute the correction to each bias in the network:
  (9) $$\Delta b_{i}=\frac{1}{\beta }(\rho (u_{i}^{\beta })-\rho (u_{i}^{0}))$$
  and adjust the biases using *b*_*i*_ ← *b*_*i*_ + αΔ*b*_*i*_.

This can be repeated on as many training examples as desired. Training can be done on batches by computing Δ*W*_*ij*_ and Δ*b*_*i*_ for each input-output pair in the batch, and correcting using the averages of these values. Note that the correction to a weight is computed using only the activations of neurons it directly affects, and the correction to a bias is computed using only the activation of the neuron it directly affects. This contrasts with backpropagation, where to correct a weight or bias *l* layers from the output it is necessary to know the activations, derivatives, and weights of all neurons between 0 and *l* − 1 layers from the output.

### 2.2. Vanishing Gradient Problem

Vanishing gradients are problematic because they reduce a network's rate of training and could be difficult to represent in neuromorphic analog hardware due to limited bit depth. For instance, in a neuromorphic hardware system with 8-bit communications and weight storage, weight corrections near the input layer of the network may be smaller than the least significant bit (due to gradient attenuation) causing those small weight changes to be lost entirely.

The vanishing gradient problem is familiar in the context of conventional feedforward networks, where techniques, such as the weight initialization scheme in Glorot and Bengio (2010), the use of activation functions with derivatives that do not lead to output saturation (Schmidhuber, 2015), and batch normalization (Ioffe and Szegedy, 2015) have been effective at overcoming it. However, in the context of the networks trained in Scellier and Bengio (2016), the vanishing gradient problem persists even when the former two techniques are used. To our knowledge batch normalization has not been used in the context of equilibrium propagation; however, it seems unlikely to be biologically-plausible.

### 2.3. Small-World Networks

The topology presented in this paper is inspired by small-world networks described in Watts and Strogatz (1998). In that paper the authors consider ring lattice networks which are highly-cliquish in the sense that the neighbors of a vertex, defined as the set of vertices with which the vertex shares an edge, are very likely to share edges with one-another. The typical distance between a pair of vertices in such a network, defined as the minimum number of edges that must be traversed in order to move between them, tends to be large as well. The authors demonstrate that when each edge in the network is randomly re-wired with some probability *p*, the typical distance is reduced at a much higher rate than the cliquishness as *p* increases, and one can thereby create a network with a high degree of cliquishness yet a short typical path length between vertices. They call such a network a small-world network.

Since in our context networks with multilayer feedforward topology have gradients that attenuate by a multiplicative factor of distance from the output layer, it seems reasonable to expect attenuation to be reduced when the typical number of connections between a given neuron and the output layer is reduced. Based on this we were motivated to explore the small-world-inspired topology described in subsequent sections as a way to reduce the typical number of connections between a pair of neurons and therefore between a neuron and the output layer, while largely preserving the layered structure of a network. It is worth noting that multilayer feedforward topology is not a perfect analogy for a circular lattice network because it lacks connections within layers and is therefore not cliquish based on the definition used in Watts and Strogatz (1998); nonetheless, we have seen empirically that our small-world-inspired topology does mitigate the vanishing gradient problem and generally improve network performance.

### 2.4. Related Work

There is a variety of work exploring layer-skipping connections in the context of deep neural networks. He et al. (2015) uses layer-skipping identity mappings to train deep convolutional neural networks through backpropagation and demonstrates that this leads to substantial performance improvements. Srivastava et al. (2015a) proposes a similar method where the transformation applied by each layer is a superposition of a nonlinear activation function and an identity mapping where the relative weights of the two components can change based on how saturated a layer's activation functions are, and demonstrates that with this method deep networks can be trained more-effectively using stochastic gradient descent with momentum. These approaches differ from ours in that they are not random and have not been applied in the context of Equilibrium Propagation, but might be effective for similar reasons as our approach. Xiaohu et al. (2011) uses an approach similar to ours to convert a multilayer feedforward network into one with a small-world-like topology and finds that doing so improves performance on a function approximation task using backpropagation. Krishnan et al. (2019) demonstrates that deep neural networks can be pruned by starting with a small-world-style network rather than a network with an excessive number of parameters without significant compromises to final model accuracy or sparsity, thereby reducing the memory requirements of doing so. The successes seen in these other works suggest that layer-skipping connections can make neural networks train more-effectively. To our knowledge our work is the first that applies layer-skipping connections to mitigate the vanishing gradient problem in networks trained using Equilibrium Propagation.

There are various approaches to mitigating the vanishing gradient problem that do not use layer-skipping connections. Ioffe and Szegedy (2015) presents a practical method for normalizing inputs to each layer of a network throughout training so that the distribution of activation values from layers is roughly uniform throughout the network, and finds that this increases performance, providing regularization and makes the weight initialization less-important. We do not use this method in our experiments because it seems unlikely to be biologically-plausible. Glorot and Bengio (2010) presents a weight initialization scheme for deep neural networks which makes train faster and more-uniformly across layers; we use this initialization scheme in our experiments.

Bartunov et al. (2018) explores the present state of biologically-motivated deep learning. This describes attempts at biologically-plausible deep learning other than Equilibrium Propagation, and contextualizes the classification error on MNIST that we see with networks trained using Equilibrium Propagation and the small-world-inspired topology we introduce in this paper. Bengio et al. (2015) discusses the criteria a biologically-plausible network would need to satisfy, and provides context to our attempt to solve the vanishing gradient problem in a biologically-plausible way.

Pedroni et al. (2019) discusses various means of storing weights in memory in the context of neuromorphic implementations of spike timing dependent plasticity, which is similar to equilibrium propagation in that it is a biologically-inspired learning algorithm that performs weight updates using local information.

## 3. Implementation

We implemented^1^ the Equilibrium Propagation framework described in Scellier and Bengio (2016) using Pytorch (Paszke et al., 2019). Like the networks in Scellier and Bengio (2016), our networks are continuous Hopfield networks with a hard sigmoid activation function

σ(x)=Max{0,Min{x,1}}

and squared-error cost function with no regularization term

$$C=||y-y_{d}|{|}_{2}^{2},$$

where **y** is the network's output and **y**_*d*_ is the target output. As described in section 5.1 of Scellier and Bengio (2016), we numerically approximate the dynamics described by Equation (5) using a forward Euler approximation with step size ϵ, *N*_*free*_ iterations of the free phase of training and *N*_*weakly*−*clamped*_ iterations of the weakly-clamped phase of training.

We use two performance-enhancing techniques that were used in Scellier and Bengio (2016): we randomize the sign of β before training on each batch, which was found in the original paper to have a regularization effect, and we use persistent particles, where the state of the network after training on a given batch during epoch *n* is used as the initial state for that batch during epoch *n* + 1. Persistent particles are useful when simulating equilibrium propagation on a digital computer because they allow the network to start an epoch closer to the equilibrium state, thereby reducing the number of iterations of the forward Euler approximation that are necessary to get sufficiently close to equilibrium. Note that this technique leads to higher error rates early in training than would be present with a more-thorough approximation of the differential equation. For purposes of computational efficiency we compute training error throughout training by recording the classification error on each training batch prior to correcting the network's parameters, and we compute the test error by evolving to equilibrium and evaluating classification error for each batch in the test dataset after each full epoch of training. In some of our trials (e.g., **Figure 4**) this approach causes the training error to exceed the test error early on in training because the network has undergone only part of an epoch of training prior to evaluating error on each training batch, but a full epoch prior to evaluating error on each test batch.

In all networks we use the weight initialization scheme in Glorot and Bengio (2010) for the weights of interlayer connections; weights connecting a pair of layers with *n*_1_ and *n*_2_ neurons are taken from the uniform distribution $U\left[-\sqrt{\frac{6}{n_{1}+n_{2}}},\sqrt{\frac{6}{n_{1}+n_{2}}}\right]$. We have found empirically that for new connections added in our topology, for a network with *N* layers it works reasonably well to draw all initial weights from *U*[−*a, a*] where $a=\frac{1}{N}\overset{N-1}{\underset{i=1}{\sum }}\sqrt{\frac{6}{n_{i}+n_{i+1}}}$ and *n*_*i*_ denotes the number of neurons in layer *i*.

### 3.1. Multilayer Feedforward (MLFF) Topology

**Figure 1:**
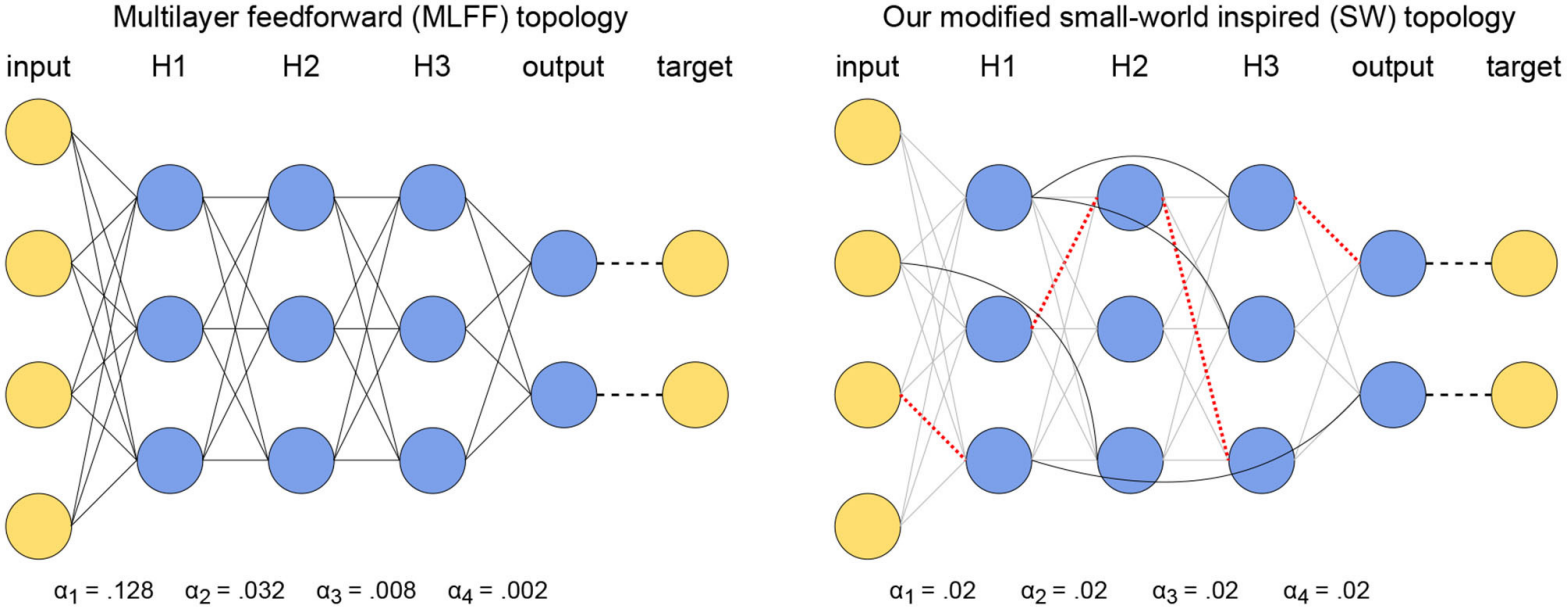
Illustration of our topological modifications to mitigate the vanishing gradient problem while using a global learning rate. **(Left)** A network with MLFF topology as tested in Scellier and Bengio (2016). Observe that the learning rate increases by a factor of 4 each time the distance from the output increases by one layer. **(Right)** A network with SW topology, where a subset of connections have been replaced by random layer-skipping connections and per-layer learning rates have been replaced by a single global learning rate. Red dotted lines denote removed connections and solid black lines denote the layer-skipping connections replacing them.

The purpose of this paper is to address the vanishing gradient problem that is present in networks with a multilayer feedforward (MLFF) topology in Scellier and Bengio (2016). Therefore, we have done a variety of trials on networks with MLFF topology to provide points of reference. The MLFF topology is illustrated to the left in Figure 1; it consists of layers of neurons with connections between every pair of neurons in adjacent layers, no connections within layers, and no connections between neurons in non-adjacent layers.

In some trials we use a single global learning rate α, and in other trials we use per-layer rates individually tuned to counter the vanishing gradient problem. The latter case entails *N* + 1 unique learning rates α_*i*_, *i* = 1, …, *N* + 1 for a network with *N* hidden layers, where the weights connecting layers *i* and *i* + 1 and the biases in layer *i* have learning rate α_*i*_.

### 3.2. Small-World Inspired (SW) Topology

We generate a network with small-world inspired (SW) topology by first starting with a MLFF topology as described above in section 3.1, then applying an algorithm similar to the one described in Watts and Strogatz (1998) to randomly replace^2^ existing connections by random layer-skipping connections with some probability *p*. This is done using Algorithm 1, and the resulting SW topology is illustrated to the right in Figure 1. In all of our trials networks with SW topology are trained using a single global learning rate α.

**Algorithm 1:**
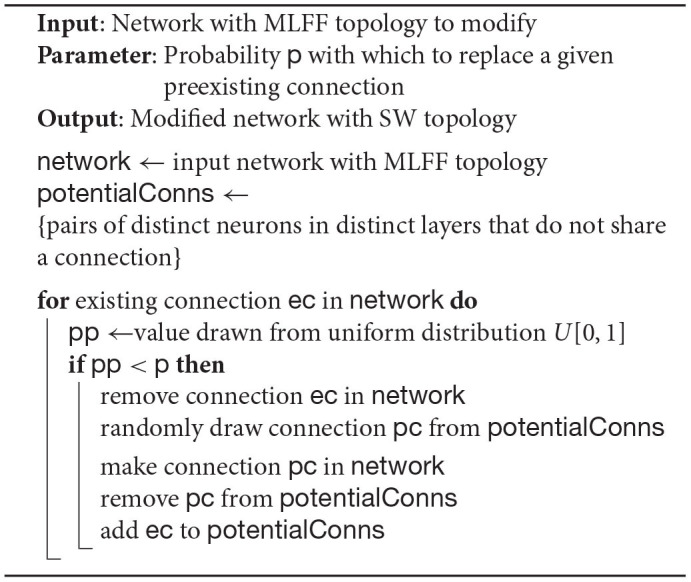
**Algorithm to generate SW topology starting with a network with MLFF topology**.

## 4. Results

### 4.1. Evaluation on MNIST Dataset

Here we compare the behavior of networks with the SW topology presented here to those with the MLFF topology used in Scellier and Bengio (2016) when training on the MNIST dataset. We are using this dataset because it allows us to reproduce and extend the trials in Scellier and Bengio (2016), and because it is non-trivial to effectively train on yet small enough to allow trials to complete in a reasonably-short amount of time.

#### 4.1.1. Classification Error

**Figure 2:**
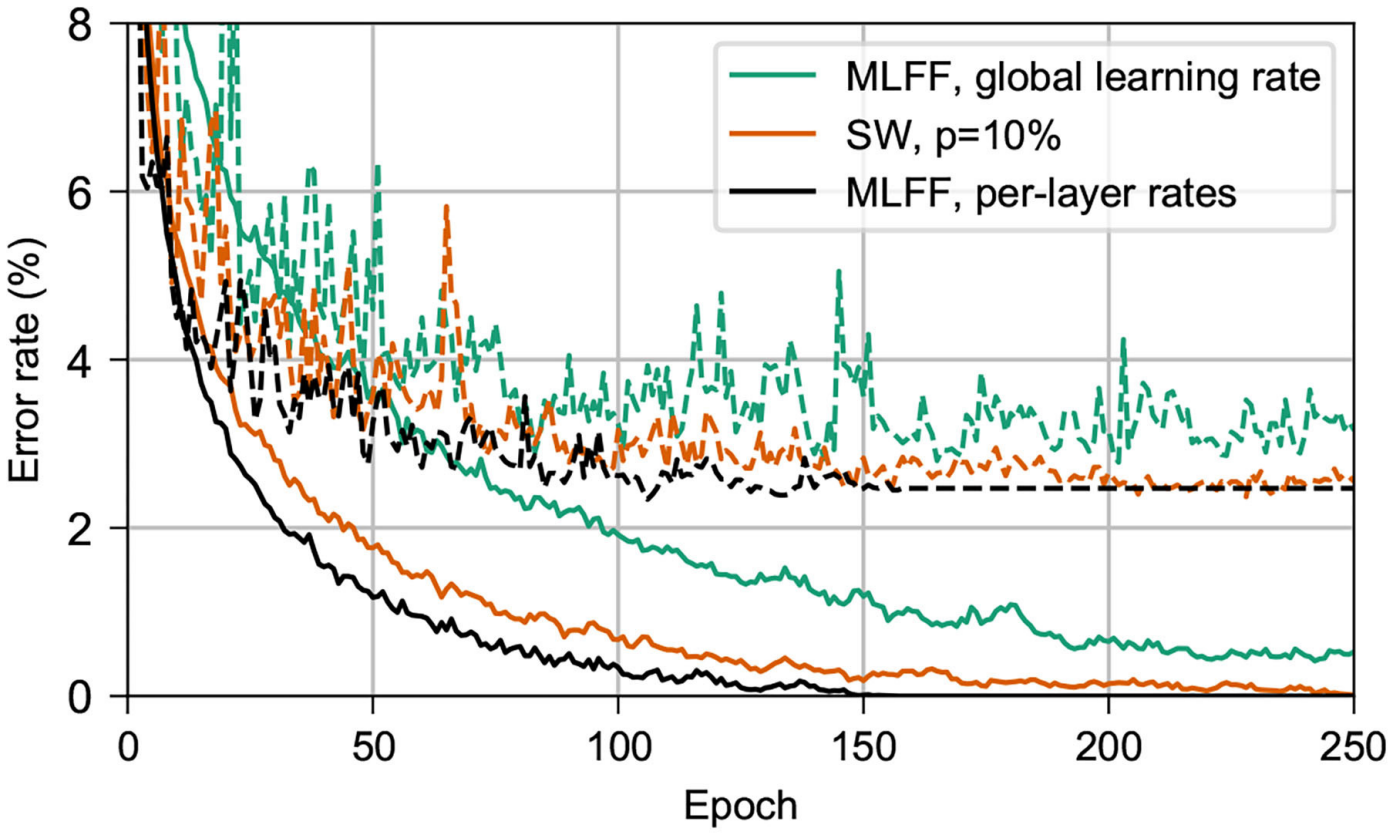
Performance on MNIST of networks with 3 500-neuron hidden layers. Dashed lines show the test error and solid lines show the training error. In black is a MLFF network with per-layer rates individually tuned to counter the vanishing gradient problem. In green is the same MLFF network but with a single global learning rate. In orange is a network with SW topology, *p* = 10%. Observe that the network with our topology trains almost as quickly as a network with per-layer rates, and significantly more-quickly than a network with a single learning rate.

Figure 2 shows the results of comparing the classification error on MNIST of a network with SW topology to that of a MLFF network with individually-tuned per-layer learning rates, as in Scellier and Bengio (2016), and to that of a MLFF network with a single global learning rate. For all networks we use 3 500-neuron hidden layers, ϵ = 0.5, β = 1.0, 500 free-phase iterations, eight weakly-clamped-phase iterations, and train for 250 epochs. For the SW network we use *p* = 10% and a global learning rate α = 0.02. For the MLFF network with per-layer rates we use learning rates α_1_ = 0.128, α_2_ = 0.032, α_3_ = 0.008, and α_4_ = 0.002. For the MLFF network with a single global learning rate, we use learning rate α = 0.02.

We find that both the SW network and the MLFF network with per-layer rates significantly outperform the MLFF network with a single global learning rate during the first 250 epochs of training. The SW network achieves training and test error rates similar to those of the MLFF network with per-layer rates, albeit after around 100 additional epochs of training.

#### 4.1.2. Training Rates of Individual Pairs of Layers

**Figure 3:**
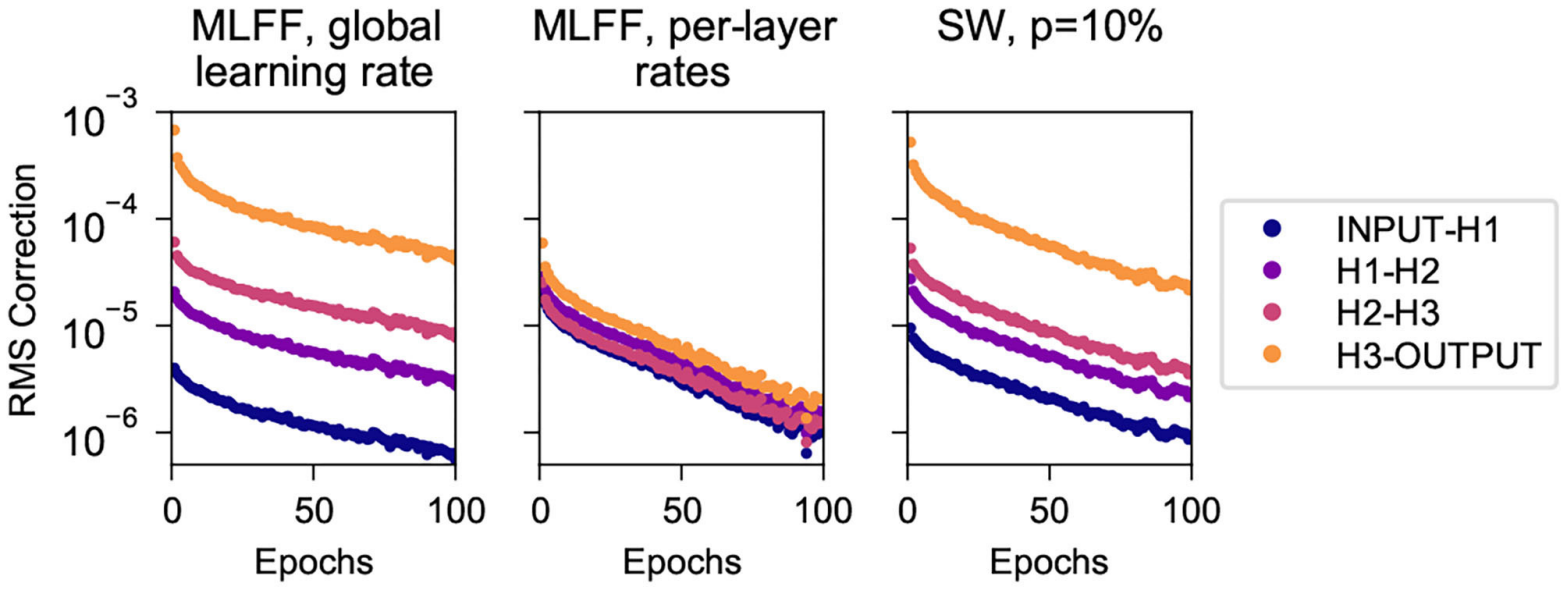
Root mean square corrections to weights in different layers while training on MNIST, for networks with 3 500-neuron hidden layers. **(Left)** A MLFF network with a single global learning rate. **(Center)** A MLFF network with per-layer rates individually tuned to counter the vanishing gradient problem. **(Right)** A network with SW topology, *p* = 10%. Observe that the correction magnitudes attenuate significantly with depth in the MLFF network with a single global learning rate, and that a network with SW topology reduces the severity of the issue, albeit less-effectively than individually tuning a learning rate for each layer. It can be seen that the RMS correction of H3-OUTPUT differs significantly from that of deeper weights; we have observed more-generally that when we transition to a SW topology the weights closest to the output train significantly faster than other weights while the training rates of deeper weights cluster together (this can also be seen in Figure 4).

Here we consider the first 100 epochs of the trials described in section 4.1.1 above and track the root-mean-square correction to weights connecting each pair of adjacent layers. Figure 3 shows these values for the three network topologies, recorded after each batch and averaged over each epoch.

We can clearly see the vanishing gradient problem for the MLFF network with a single global learning rate (left), manifesting as attenuation with depth of the root-mean-square corrections to weights. The problem is addressed very-effectively by the use of manually-tuned per-layer learning rates (center), and is mitigated to a more-modest extent when we use SW topology with a global learning rate. It is noteworthy that the speed of training of these networks as shown in Figure 2 is commensurate with the uniformity with which their layers train as shown in Figure 3.

It can be seen that in the network with SW topology the weights connecting to the output layer train significantly faster than deeper weights, which cluster together; we have observed similar behavior in a variety of datasets and network dimensions. We suspect it has to do with the fact that output neurons connect directly to the target output, whereas because layer-skipping connections are not attached to the target output the other layers must connect indirectly through at least two connections.

#### 4.1.3. Behavior for Varying *p*

**Figure 4:**
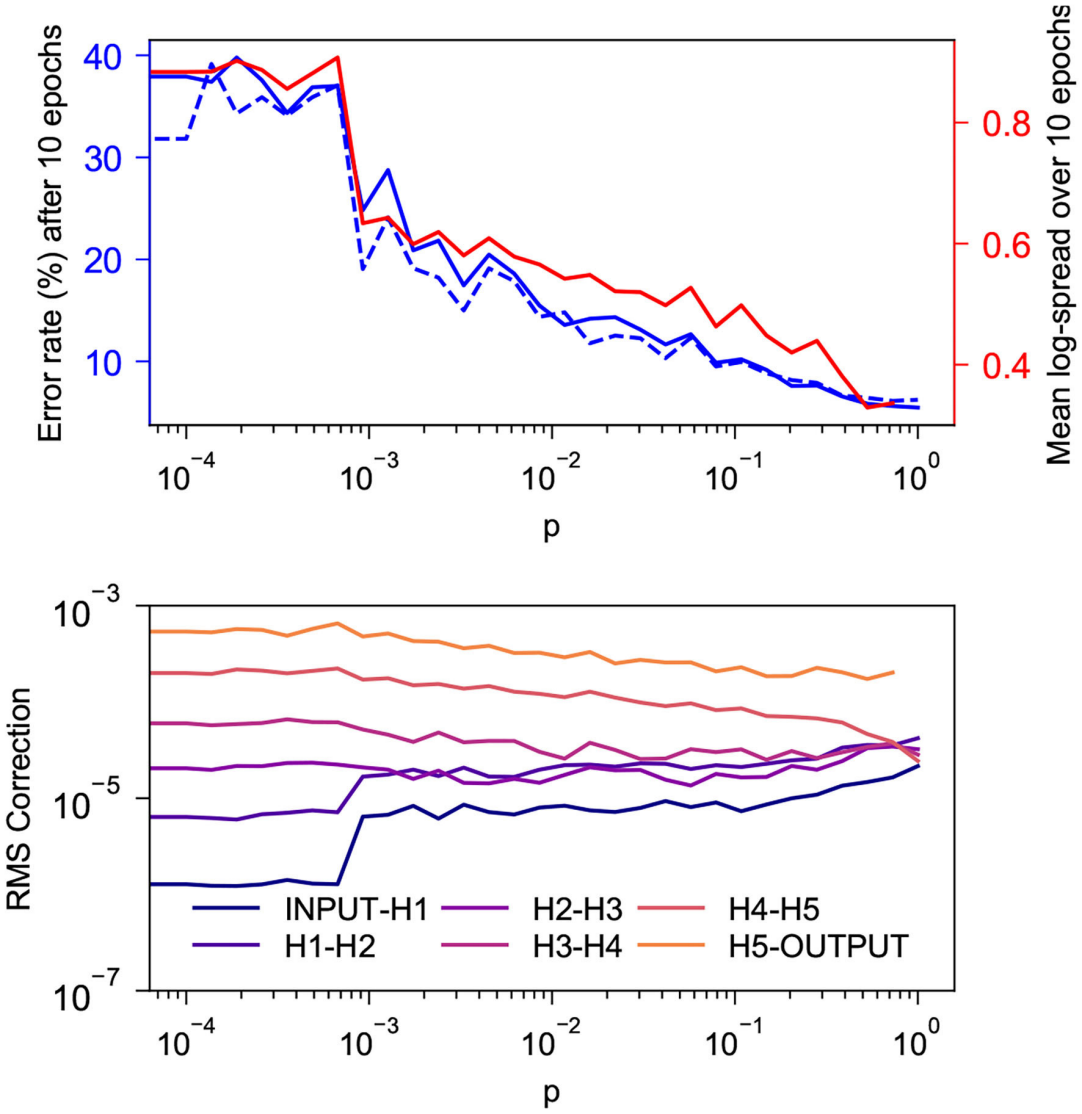
Behavior during the first 10 epochs of training on MNIST for a SW network with 5 100-neuron hidden layers for various values of *p*. **(Top)** In solid and dashed blue are the training and test error rates after 10 epochs and in red is the log-spread (Equation 10) averaged over the 10 epochs. As expected, both values decrease as *p* increases. There appears to be a strong linear correlation between the two, with coefficient of determination *r*^2^ = 0.970. This is consistent with our suspicion that mitigation of the vanishing gradient problem is the reason our topology tends to increase the rate at which layered networks train. **(Bottom)** Root mean square corrections to weights in different layers, averaged over the 10 epochs. As expected, the spread of these values decreases as *p* increases.

Figure 4 shows the behavior during the first 10 epochs of training on MNIST for a network with SW topology as *p* is increased from 0 to 0.727. The network being tested has 5 100-neuron hidden layers and is trained with α = 0.015, ϵ = 0.5, β = 1.0, 1,000 free-phase iterations and 12 weakly-clamped-phase iterations.

We see in the top graph that the training and test error rates after 10 epochs decay exponentially as *p* increases. The bottom graph indicates that the RMS corrections to weights become more-uniform with depth as *p* increases. Corrections to layers that do not connect directly to the output layer cluster closer together as *p* increases, but it appears that the corrections to weights connecting directly to the output layer train faster than deeper weights with a gap that does not appear to decrease with *p*; this is similar to the behavior seen in section 4.1.2.

To quantify the spread of the RMS corrections as a single scalar, we introduce the statistic

(10) $$\text{log-spread}=\text{Std. dev}\{{\text{log}}_{10}(w_{l}),l=1,\ldots ,N+1\}$$

where *N* denotes the number of hidden layers in a network and *w*_*l*_ denotes the root mean square magnitude of corrections to weights connecting the *l*th and *l* + 1th layers from the input, averaged over all epochs of training. In an ideal scenario, the values of *w*_*l*_ would tend to be approximately the same, leading to log-spread close to 0. Large disparities in values of *w*_*l*_, leading to a large log-spread, indicate that some layers in the network are being trained much more thoroughly than others. The log-spread is plotted in the top graph alongside the error rate, and these values can be seen to have a strong linear correlation. The training error and the log-spread have a coefficient of determination *r*^2^ = 0.970.

#### 4.1.4. Weight Correction Matrix

**Figure 5:**
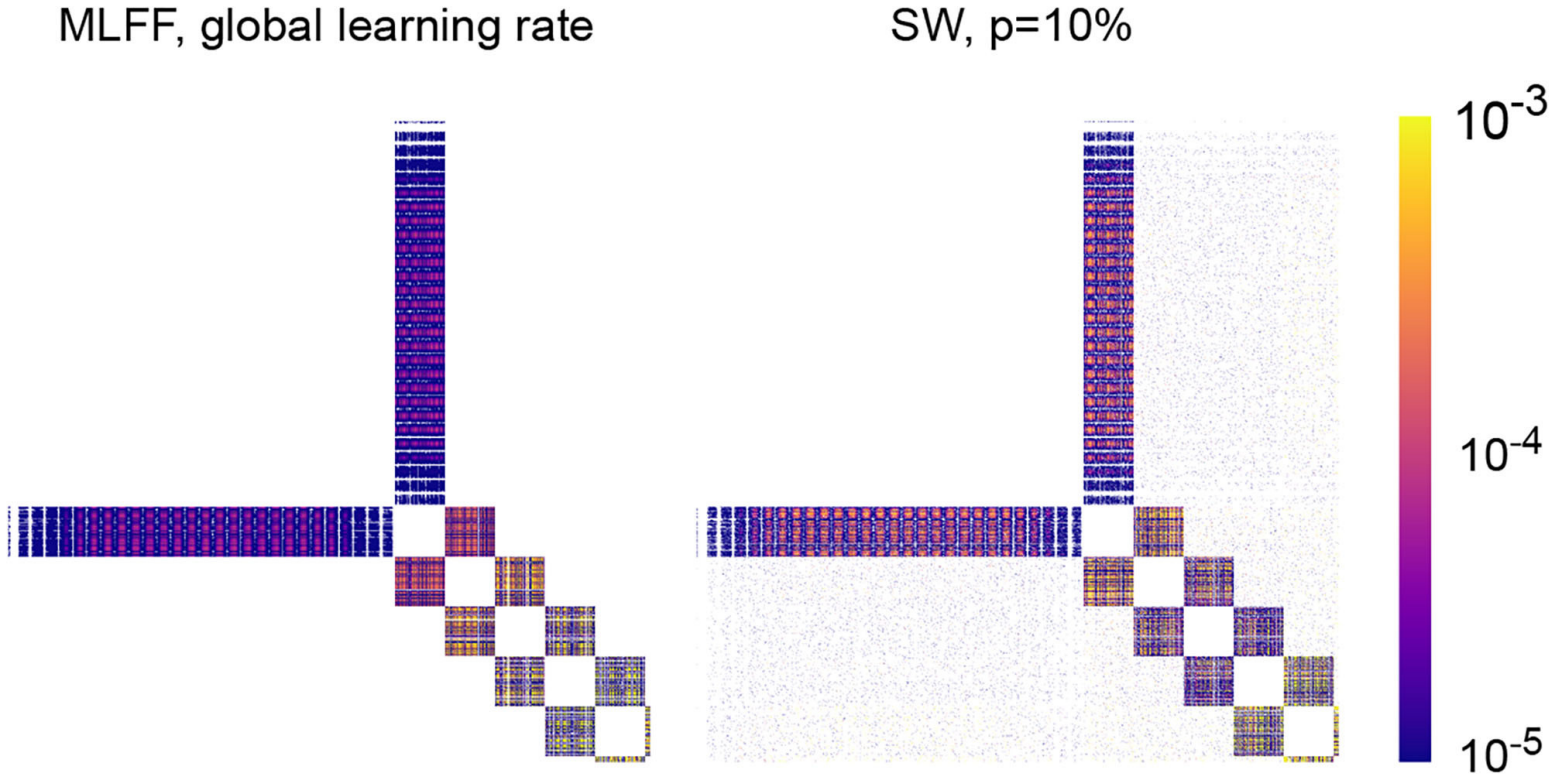
Mean absolute value correction matrix over 100 epochs for networks with 5 100-neuron hidden layers. A pixel at position (*i, j*) corresponds to the magnitude of the correction to the connection weight between neurons *i* and *j*. **(Left)** A network with MLFF topology and a single global learning rate. **(Right)** A network with SW topology, *p* = 10%. Observe that attenuation of these values with depth is less-significant in the latter than in the former.

In Figure 5 we visualize the mean weight correction matrices resulting from training a MLFF network and a SW network with 5 100-neuron hidden layers as described in section 4.1.3. Training on a batch yields a correction matrix **dW** where element **dW**_*ij*_ denotes the change to the connection weight between neurons *i* and *j*; here we have recorded a matrix with element (*i, j*) containing the average of |**dW**_*ij*_| over 100 epochs of training and displayed it as an image with the color of a pixel at position (*i, j*) encoding the magnitude of |**dW**_*ij*_| as indicated by the legend.

As expected, there is attenuation with depth to weight correction magnitudes in a MLFF network with a single global learning rate, and transitioning to a network with SW topology reduces the severity of the problem. An interesting feature of the SW matrix that is visible upon close inspection is that layer-skipping connections tend to receive larger correction magnitudes when they are closer to the output of the network. Striations can be seen in correction magnitudes, which we believe correspond to sections of images in the MNIST dataset that contain varying amounts of information; for example, for corrections connecting to the input layer, there are 28 striations which likely correspond to the 28 rows of pixels in an image of a digit, with pixels closer to the edges of the images typically blank and pixels closer to the centers of the images typically containing most of the variation that encodes a digit.

### 4.2. Evaluation on Various Datasets and Topologies

Here we evaluate the presence of the vanishing gradient problem and the effectiveness of our topology at addressing it on MNIST (LeCun and Cortes, 1998), Fashion MNIST (FMNIST) (Xiao et al., 2017), and the diabetes and wine toy datasets distributed in scikit-learn (Pedregosa et al., 2011) with various network architectures. Our results are shown in Table 1. For all of these trials we use β = 1.0 and ϵ = 0.5. While hyperparameters in these trials have not been thoroughly optimized, the trials serve to illustrate that the trends we saw on the MNIST dataset in section 4.1 hold for deeper networks and for networks training on 4 different datasets.

**Table 1:** Comparison of MLFF and SW topologies with various datasets and network architectures.

**Dataset**	**Layer sizes**	**Topology**	**L.R**.	**Iterations**	**Error (train/test)**	**Log-spread**
Diabetes	10-10-10-10-10-10-1	MLFF	0.01	1000/12	0.0326/0.0441	1.085
Diabetes	10-10-10-10-10-10-1	SW, *p* = 10%	0.01	1000/12	0.0302/0.0394	**0.557**
Diabetes	10-10-10-10-10-10-10-10-10-1	MLFF	0.01	5000/18	0.0318/0.0425	2.490
Diabetes	10-10-10-10-10-10-10-10-10-1	SW, *p* = 10%	0.01	5000/18	0.0230/0.0339	**0.442**
Wine	13-10-10-10-10-10-3	MLFF	0.01	1000/12	0.680/0.760	1.216
Wine	13-10-10-10-10-10-3	SW, *p* = 10%	0.01	1000/12	0.467/0.280	**0.689**
Wine	13-10-10-10-10-10-10-10-10-3	MLFF	0.01	5000/18	0.653/0.720	1.734
Wine	13-10-10-10-10-10-10-10-10-3	SW, *p* = 10%	0.01	5000/18	0.253/0.560	**0.471**
MNIST	784-500-500-500-10	MLFF	0.02	500/8	0.0170/0.0310	0.689
MNIST	784-500-500-500-10	SW, *p* = 10%	0.02	500/8	0.00675/0.0272	**0.545**
MNIST	784-100-100-100-100-100-10	MLFF	0.015	1000/12	0.156/0.131	0.867
MNIST	784-100-100-100-100-100-10	SW, *p* = 10%	0.015	1000/12	0.0407/0.0540	**0.450**
FMNIST	784-100-100-100-100-100-10	MLFF	0.015	1000/12	0.266/0.255	0.862
FMNIST	784-100-100-100-100-100-10	SW, *p* = 10%	0.015	1000/12	0.152/0.164	**0.484**

*Trials were run for 100 epochs with batch size 20 on MNIST and FMNIST and for 10 epochs with batch size 5 on Diabetes and Wine. Observe that the vanishing gradient problem, quantified by the log-spread (Equation 10), is significantly improved when using the SW topology over the MLFF topology. Error is given as proportion of classes incorrectly identified for MNIST, FMNIST, and Wine (classification problems) and as mean absolute value of error for Diabetes (regression problem). Values are bold for emphasis. We are trying to draw attention to the fact that the log-spread for networks with SW topology are smaller than those for equivalent networks with MLFF topology*.

We report the training and test error after 100 epochs, as well as the log-spread (Equation 10). We see that networks with SW topology, *p* = 10%, have a consistently smaller log-spread than networks with a MLFF topology and a single global learning rate. Here this associated with smaller training and test error rates, though we have seen for some less-optimized combinations of hyperparameters that the error rates do not significantly change. We have observed that all of these networks behave in ways that are qualitatively similar to the networks explored in section 4.1.

## 5. Directions for Future Research

There are several directions in which future research could be taken:

- Evaluating the effectiveness of this approach on hard datasets, such as CIFAR and ImageNet.
- Evaluating the effect of *p* on a network's test error in the long term.
- Exploring the effectiveness of a network when layer-skipping connections are used during training and removed afterwards.
- Devise and mathematically justify a weight initialization scheme for layer-skipping connections.
- Mathematically justify the empirical results we have seen when using SW topology.

## Data Availability Statement

The datasets presented in this study can be found in online repositories. The names of the repository/repositories and accession number(s) can be found at: Data and code used to generate figures: https://github.com/jgammell/eqp_paper_data; Code used to run experiments: https://github.com/jgammell/equilibrium_propagation.

## Author Contributions

JG wrote the code and ran the experiments to determine the effects of layer-skipping connections. SB analyzed the differences between equilibrium propagation and backpropagation. SN provided the guidance. AM wrote the code and assisted with layer-skipping analysis. All authors contributed to the article and approved the submitted version.

## Conflict of Interest

The authors declare that the research was conducted in the absence of any commercial or financial relationships that could be construed as a potential conflict of interest.

## Footnotes

^1^https://github.com/jgammell/equilibrium_propagation.

^2^We have done a small number of trials in which layer skipping connections are added without removing an equal number of preexisting connections, and have observed behavior similar to that resulting from using Algorithm 1.
