# Supplementary material for: Layer-Skipping Connections Improve the Effectiveness of Equilibrium Propagation on Layered Networks
Source: Front Comput Neurosci. 2021 May 17;15:627357. doi: 10.3389/fncom.2021.627357 (PMC8165608; doi:10.3389/fncom.2021.627357)
Supplement: Supplementary file 1 [file Data_Sheet_1.pdf]

TABLES

	Backpropagation	Equilibrium Propagation
Number of distinct computations	2 – computations during forwards and backwards phases are distinct	≈ 1 – hidden neurons perform same computation in both phases. Output neurons perform a similar but modified version of the same computation.
Types of connections	Unidirectional to transmit activation to shallower neighbors and error to deeper neighbors	Bidirectional to each neighbor
Memory	Space to store activation and error term for each neuron	Space to store free and weakly-clamped activations for each neuron
Order of computations	Forwards propagation phase where layers are computed from deepest to shallowest; backwards propagation phase where layers are computed from shallowest to deepest; parameter update phase	Free phase where all neurons evolve simultaneously; weakly-clamped phase where all neurons evolve simultaneously; parameter update phase
Nonlinear activation function	Yes	Yes
Derivative of nonlinear activation function	Yes	Yes
Correction computation	Corrections require dedicated circuitry unique from that implementing propagation	Corrections require dedicated circuitry unique from that implementing evolution

Table 2. Comparison of the capabilities a hardware neuron would need in order to implement backpropagation and equilibrium propagation.

A COMPARING THE COMPUTATIONAL COMPLEXITY OF EQUILIBRIUM PROPAGATION AND BACKPROPAGATION

The main motivations for using equilibrium propagation instead of an alternative machine learning technique (such as deep learning using backpropagation for training) are 1) to gain insight into the operation of the brain by developing target-based learning approaches in biologically plausible networks and 2) to develop algorithms that are more easily implemented in hardware. Below we qualitatively compare the hardware that would be needed for implementation of equilibrium propagation versus for backpropagation on a standard feedforward network to gain insight into the utility of these networks.

A.1 Requirements of equilibrium propagation

Just as in the algorithm, to implement equilibrium propagation in hardware, three different phases of hardware operation are required. In the first (free) phase, it follows from equations 2, 4 and 5 that to determine its state, the i-th neuron in a network must compute

$$\frac{\partial F}{\partial u_i} = u_i - \frac{1}{2}\rho'(u_i)[\sum_{i\neq j} W_{ij}\rho(u_j) + b_i],$$

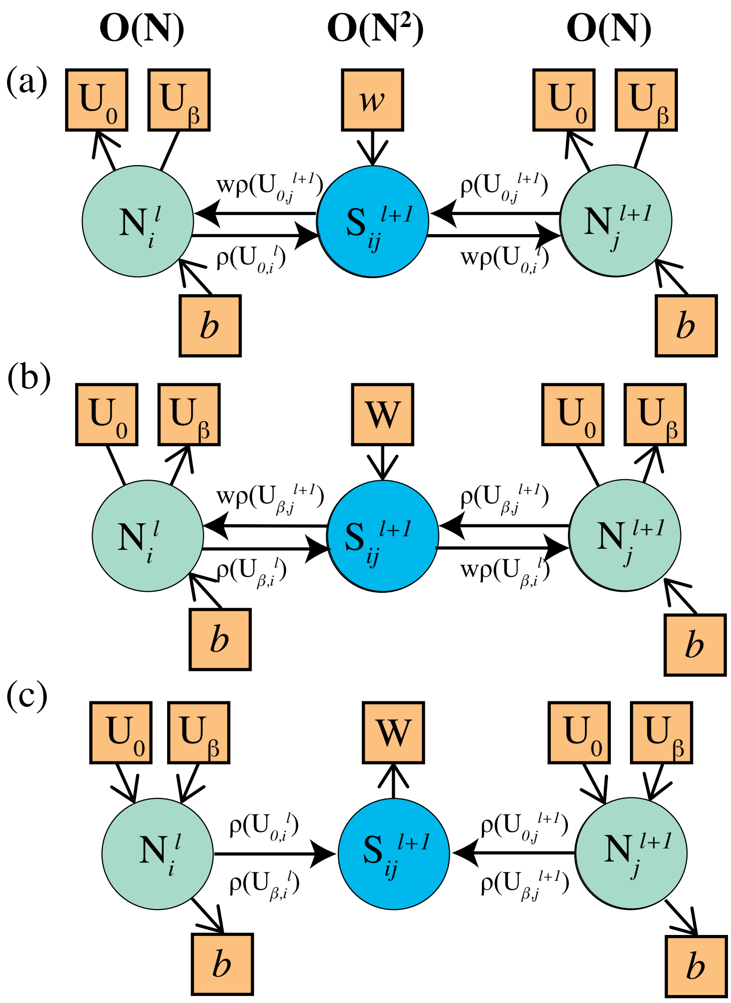

Figure 6. Illustration of the functionality needed to implement equilibrium propagation in hardware. Yellow squares indicate a value that must be stored in memory for a subsequent phase. The circles indicate (N) neuron and (S) synapse devices with the associated functions described in the text. (a) The functionality required by the neurons and synapses in the free running phase. (b) The functionality of the neurons and synapses (except output neurons) in the weakly clamped phase. (c) The functionality of the neurons and synapses in the weight and bias update phase.

plus the term $\beta(u_i - y_i^{target})$ for output neurons when using a squared-error cost function, and then integrate the result over time. Parameter correction rules are given by equations 8 and 9. This is exactly the operation of an analog leaky integrate and fire neuron, as for example implemented in the neuromorphic hardware platforms of references [Indiveri et al., 2011; Schemmel et al., 2010] among others. A qualitative diagram of potential neuron and synapse devices and their output and read/write to memory operations are shown in the diagram in figure 6 (a). At each neuron N_i^l the value of $U_{i,0}^{l+1}$ is written to memory and the nonlinear function ρ is applied before sending to the synapse device, where it is multiplied by the weight w_{ij}^{l+1} which is read from memory by the synapse device. These weighted outputs are summed at the input of the next neuron device (N_j^{l+1}) and added to a bias value b_j^{l+1} that is read from memory to generate U_{l+1}.

In the second (weakly-clamped) phase, shown in figure 6 (b), the operation of the hardware is exactly the same as in (a), with the value U_β written to memory at each neuron. Not shown in figure 6 is the functionality at the output neurons which are weakly clamped and have a new function in this phase.

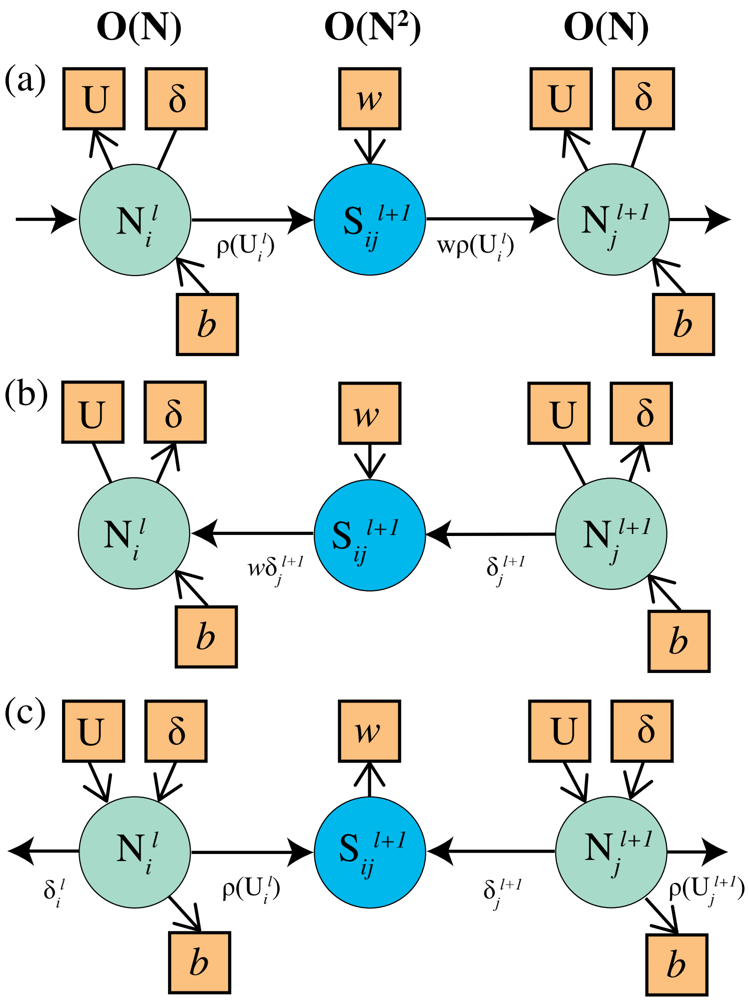

Figure 7. Illustration of the functionality needed to implement backpropagation in hardware. Yellow squares indicate a value that must be stored in memory for a subsequent phase. The circles indicate (N) neuron and (S) synapse devices with the associated functions described in the text. (a) The functionality required by the neurons and synapses in the forward pass phase. (b) The functionality of the neurons and synapses (except the last layer of neurons) in the backpropagation phase. (c) The functionality of the neurons and synapses in the weight and bias update phase.

Finally, in the third phase, the weights and biases are updated as shown in figure 6 (c). At each neuron device the values of U_0 and U_β are read from memory and $\rho(U_0)$ and $\rho(U_\beta)$ are calculated. At the synapse device, the computation of equation 8 is performed using these values from the pre- and post- synaptic neurons to calculate the weight update Δw, and the value of the weight in memory is updated to $w + \alpha\Delta w$. Similar updates are applied to the bias b at every neuron according to equation 9. As shown at the top of 6, for N such neurons per layer, there will be N^2 synapses.

A.2 Requirements of backpropagation

Backpropagation is an algorithm for training networks using gradient descent. It is most typically applied to feedforward neural networks, in which the activation value of a neuron i in layer l is given by

$$\rho(u_i^l) = \rho(\sum_j W_{ij}^l u_j^{l-1} + b_i^l).$$

This is very similar to the free running situation in equilibrium propagation, with the main difference being that the connections are unidirectional. The qualitative implementation of this inference phase in hardware is shown in Fig. 7 (a). Using backpropagation, the parameters are then updated by computing error correction terms δ_i^l for each neuron i in layer l; for the output layer L the correction is

$$\delta_i^L = \rho'(u_i^L)(\rho(u_i^L) - y_i^{target})$$

and for deeper layers it is

$$\delta_i^l = \rho'(u_i^l) \sum_j W_{ij}^{l+1} \delta_j^{l+1}.$$

The implementation of this in hardware is shown in figure 7 (b) (excluding layer L). Note that the data is now moving in the opposite (backwards) direction, and unlike in the case of equilibrium propagation, the functions implemented by the neurons are entirely different to the operation in the forward phase shown in (a). In a final phase, weights are corrected using

$$\Delta W_{ij}^l = \rho(u_i^{l-1})\delta_j^l$$

and biases using

$$\Delta b_i^l = \delta_i^l.$$

This is shown in figure 7 (c).

A.3 Comparison

The most-significant difference between the algorithms is that in equilibrium propagation, the free and weakly-clamped phases of training are identical for most neurons and the weakly-clamped phase requires only slight modification to output neurons, whereas in backpropagation these phases demand significantly-different functionality from essentially all neurons. There are two other differences that we do not believe to be significant in terms of ease of implementation in hardware. One is that in equilibrium propagation each pair of neurons is joined by a bidirectional synapse, whereas in backpropagation each pair is joined by two unidirectional synapses; we expect both cases to be equally easy to implement. The other is that in equilibrium propagation, each neuron must remember its equilibrium state after the free phase while it executes the weakly-clamped phase; since backpropagation implies a state variable for the activation and error term of each neuron, the memory requirement of each neuron should be the same in both cases. For a hardware implementation, the need for distinct free and weakly-clamped phases (temporally non-local credit assignment) significantly reduces the advantages associated with the spatially local credit assignment. Recently there has been new work that indicates that the algorithm can be modified to eliminate the need for both phases [Ernoult et al., 2020]. This would significantly reduce the memory requirements of the algorithm. Various characteristics of both algorithms are compared side-by-side in table 2.
